# Comparative transcriptome profiling reveals the importance of *GmSWEET15* in soybean susceptibility to *Sclerotinia sclerotiorum*

**DOI:** 10.3389/fmicb.2023.1119016

**Published:** 2023-01-26

**Authors:** Kunqin Xiao, Kaibin Qiao, Wenjing Cui, Xun Xu, Hongyu Pan, Fengting Wang, Shoudong Wang, Feng Yang, Yuanhu Xuan, Anmo Li, Xiao Han, Zhuojian Song, Jinliang Liu

**Affiliations:** ^1^College of Plant Sciences, Jilin University, Changchun, China; ^2^Key Laboratory of Soybean Molecular Design Breeding, Northeast Institute of Geography and Agroecology, Chinese Academy of Sciences, Changchun, China; ^3^College of Plant Protection, Shenyang Agricultural University, Shenyang, China

**Keywords:** *GmSWEET15*, *Sclerotinia sclerotiorum*, transcriptome, gene co-expression network, susceptibility

## Abstract

Soybean sclerotinia stem rot (SSR) is a disease caused by *Sclerotinia sclerotiorum* that causes incalculable losses in soybean yield each year. Considering the lack of effective resistance resources and the elusive resistance mechanisms, we are urged to develop resistance genes and explore their molecular mechanisms. Here, we found that loss of *GmSWEET15* enhanced the resistance to *S. sclerotiorum*, and we explored the molecular mechanisms by which *gmsweet15* mutant exhibit enhanced resistance to *S. sclerotiorum* by comparing transcriptome. At the early stage of inoculation, the wild type (WT) showed moderate defense response, whereas *gmsweet15* mutant exhibited more extensive and intense transcription reprogramming. The *gmsweet15* mutant enriched more biological processes, including the secretory pathway and tetrapyrrole metabolism, and it showed stronger changes in defense response, protein ubiquitination, MAPK signaling pathway-plant, plant-pathogen interaction, phenylpropanoid biosynthesis, and photosynthesis. The more intense and abundant transcriptional reprogramming of *gmsweet15* mutant may explain how it effectively delayed colonization by *S. sclerotiorum*. In addition, we identified common and specific differentially expressed genes between WT and *gmsweet15* mutant after inoculation with *S. sclerotiorum*, and gene sets and genes related to *gmsweet15*_24 h were identified through Gene Set Enrichment Analysis. Moreover, we constructed the protein–protein interaction network and gene co-expression networks and identified several groups of regulatory networks of *gmsweet15* mutant in response to *S. sclerotiorum*, which will be helpful for the discovery of candidate functional genes. Taken together, our results elucidate molecular mechanisms of delayed colonization by *S. sclerotiorum* after loss of *GmSWEET15* in soybean, and we propose novel resources for improving resistance to SSR.

## Introduction

Soybean is an important oilseed and cash crop that is commonly cultivated for its rich protein and lipid content (Diers et al., 2018). In the past 10 years, the average annual soybean production in China has reached approximately 15 million tons. However, agricultural production of soybeans is frequently threatened by various diseases, including sclerotinia stem rot (SSR), which is a devastating plant fungal disease caused by *Sclerotinia sclerotiorum* that can damage soybean at the seedling, adult, and flowering stages. SSR mainly affects the upper parts of soybean plants and thereby affects quality and yield. In epidemic years, it can cause approximately 20%–30% reduction in soybean annual yield, with severely affected cultures experiencing 50% to complete loss (Wang et al., 2019d). The direct economic loss caused by SSR in the United States is approximately $200 million per year (Bolton et al., 2006). *Sclerotinia sclerotiorum* is a notorious necrotrophic phytopathogen that occurs in soybean cultures globally (Derbyshire et al., 2017), however, its epidemic mechanisms remain unclear, which limits the success of prevention and control measures (Hibbett et al., 2007). In recent years, SSR incidence increased, thus posing a considerable threat to soybean production. The mechanisms underlying soybean resistance to *S. sclerotiorum* remain unclear, and only few resistance resources have been explored so far.

To counteract pathogen infection, plants have evolved complex immune systems, including pathogen/microbe associated molecular patterns (P/MAMPs) triggered immunity (PTI; Wang et al., 2019d), effector protein triggered immunity (ETI; De Schutter and Van Damme, 2015; Thieffry et al., 2022), systemic acquired immunity (SAR), and complicated quantitative disease resistance (QDR). PTI acts as the first layer of defense, activating a series of immune responses through the sensing of PAMPs by plasma membrane-localized receptors, including the expression of defense genes, activation of MAPK, production of reactive oxygen species (ROS), callose deposition, and calcium influx (Wang et al., 2019d). In response to the evolution of pathogenic effectors, plants have evolved resistance (R) proteins that activate ETI through direct or indirect recognition of specific effector proteins, i.e., Avr proteins (Ngou et al., 2021; Yuan et al., 2021). In addition, plants also have a set of acquired immune mechanism termed SAR. These response are stimulated at the infection site, and distal tissues subsequently become resistant to potential pathogens through various mechanisms (Ding and Ding, 2020). QDR also occurs in plants when disease symptoms are mild but do not vanish easily. This type of resistance reduces selective pressure during pathogen evolution and is therefore generally considered more durable. QDR is typically provided by quantitative trait loci (QTL), controlled by one or multiple genes, or by multiple gene interactions (Corwin and Kliebenstein, 2017). More importantly, QDR seems to plays a more reliable role in the fight against broad host-range necrotrophic phytopathogens, such as *S. sclerotiorum*.

In recent years, a number of SSR resistance genes have been identified, including ectopic expression of polygalacturonase inhibitor protein (*OsPGIP2*) in rapeseed, which enhances resistance to *S. sclerotiorum* at the seedling and maturity stages (Wang Z. et al., 2018). Similarly, overexpression of AtPGIP1 in *Arabidopsis* reduces colonization ability of *S. sclerotiorum* (Wei et al., 2022). The kinases MPK3 and MPK4 of the MAPK signaling pathway and transcription factor WRKY33 play important roles in the resistance of rapeseed *S. sclerotiorum* (Wang et al., 2009, 2014, 2019c). BnMPK4-BnMKS1-BnWRKY33 exists in a nuclear localized complex that regulates resistance to *S. sclerotiorum*, which is most likely associated with the activation of salicylic acid (SA)- and jasmonic acid (JA)-mediated defense response and inhibition of H_2_O_2_ accumulation (Wang et al., 2014). Calcium signaling pathways are also important in plant immunity, and Ca^2+^ influx into the cytoplasm is an early responses of plants sensing pathogenic invasion. Cyclic nucleotide gated ion channels (CNGCs) in plants can generate Ca^2+^ signals and promote elevated Ca^2+^ levels in the cytoplasm in response to pathogen signals (Ma and Berkowitz, 2011). Several CNGCs have been reported to regulate the resistance to *S. sclerotiorum*, whereas several others negatively affect such resistance (Saand et al., 2015a,b). Similarly, other regulatory factors of calcium signaling positively regulate the resistance to *S. sclerotiorum* (Wang et al., 2015; Rahman et al., 2016; Wang et al., 2016). Many hormones are involved in signal transduction in plant defense responses to pathogens, such as JA, SA, ET, ABA, and growth hormones. Such hormones act as signaling molecules that transmit sensory signals which activate complex defense responses. In *Arabidopsis*, the JA/ET signaling pathway can positively regulate resistance to *S. sclerotiorum*, and ABA positively regulates JA biosynthesis. *Arabidopsis* mutants *aba3-2*, *abi1-1*, and *abi2-1* are defective in ABA synthesis and exhibit complete loss of resistance to *S. sclerotiorum* (Guimaraes and Stotz, 2004; Perchepied et al., 2010). Several studies have identified QTLs associated with resistance to *S. sclerotiorum* (Wei et al., 2016; Wu et al., 2016; Wen et al., 2018). One study identified 39 candidate SSR resistance genes at three QTLs, and among these, the gene encoding ethylene response transcription factor 73 (*ERF73*, *BnaC06g24360D*) was considered a promising candidate for disease resistance (Wu et al., 2016). A different study identified 28 loci for a number of candidate SSR resistance genes, which interestingly were associated with different biological functions, and among these, two transferase-related genes were induced within 12 h after inoculation, which encoded acyltransferases (*Glyma.04G198000*) and a UDP-glucosyltransferase (*Glyma.16G158100*) involved in secondary metabolic biosynthesis (Wen et al., 2018). Despite successful identification of numerous SSR resistance genes, their resistance mechanisms remain to be further defined and, more importantly, SSR resistance genes are far from being comprehensively characterized.

Members of the Sugars Will Eventually be Exported Transporter (SWEET) family which possessing seven transmembrane helices can mediate the bidirectional transport of carbohydrates across membranes (Chen et al., 2012; Yuan and Wang, 2013; Eom et al., 2015). Previous studies have shown that SWEETs play crucial roles in plant growth, development, and responses to biological stress processes, such as phloem loading for long distance sugar transport, hormone transport, pollen development, fruit development, seed filling, plant-pathogen interactions, and plant-microbe interactions (Moriyama et al., 2006; Wellmer et al., 2006; Micali et al., 2008; Yuan et al., 2009; Chen et al., 2010; Ayre, 2011; Seo et al., 2011; Sosso et al., 2015; Kanno et al., 2016; Gao et al., 2018). The involvement of SWEETs in pathogen-host interactions is currently best understood with regard to rice OsSWEET11, which provides nutrients to the rice leaf blight pathogen, when *Xanthomonas oryzae* pv. *oryzae* (*Xoo*) infects rice, transcription activator-like effectors (TALEs) regulates their transcriptional expression by binding to a cis-acting element in the *SWEETs* promoters, thereby accelerating the transfer of sugars from rice to *Xoo* and promoting pathogen growth and colonization (Chu et al., 2006; Yang et al., 2006; Gao et al., 2018; Xue et al., 2022). Expression of *AtSWEET2* localized in vesicles was significantly induced in *Arabidopsis thaliana* upon infestation with *Pythium irregulare*, and *AtSWEET2* mutants showed reduced resistance to *P. irregulare* (Chen et al., 2015). IbSWEET10 of sweet potato is localized at the plasma membrane, and overexpression of IbSWEET10 in plants resulted in reduced sucrose content and increased resistance to *Fusarium oxysporum* (Li et al., 2017; Xue et al., 2022). However, no studies on the role of SWEETs with regard to the interactions between *S. sclerotiorum* and its host are available.

Fifty-two *SWEETs* have been identified in the soybean genome (Patil et al., 2015), however, many of their functions remain unexplored. Both soybean (*Glycine max*) *GmSWEET15a* and *GmSWEET15b* belong to branch III of the *SWEETs*. Previous studies have shown that these genes are highly expressed in the endosperm during the cotyledon stage. Knockout of *GmSWEET15* in soybean results in delayed embryo development and endosperm persistence, leading to severe seed abortion. In addition, the embryonic sugar content is markedly reduced in these soybean knockout mutants, suggesting that the GmSWEET15 is essential for embryo development in soybean by mediating the export of sucrose from the endosperm to the embryo during seed development (Wang et al., 2019b). However, it is unclear whether GmSWEET15 also exerts other functions, particularly regarding those involved in resistance to SSR.

Here, we first illuminated that loss of *GmSWEET15* enhanced resistance to *S. sclerotiorum*, and then we explored the molecular mechanisms of the *gmsweet15* mutant enhancing the resistance to *S. sclerotiorum* by comparing transcriptome. Our comparative analyses revealed that the resistance phenotype of *gmsweet15* mutant may be mainly related to the dynamic transcriptional reprogramming response during the early stage of inoculation with *S. sclerotiorum*. In addition, we identified a series of biological processes and functional genes that may play key roles in *gmsweet15* mutant responses to *S. sclerotiorum* infection. Together, this study revealed the molecular mechanism enhanced soybean resistance to *S. sclerotiorum* in *gmsweet15* mutant and proposes numerous candidate functional genes for elevating resistance to SSR.

## Materials and methods

### Plant materials and *Sclerotinia sclerotiorum* inoculation

*Arabidopsis thaliana* (Col-0) plants maintained at 16/8 h light/darkness, with 22°C during the day and 18°C at night, and soybean plants (Williams 82 and *gmsweet15-1*/Williams 82 mutant) maintained at 16/8 h light/darkness, with 30°C during the day and 25°C at night, were grown in a greenhouse. The *gmsweet15-1* mutant was donated by Professor Huixia Shou, College of Life Sciences, Zhejiang University.

The *S. sclerotiorum* wild-type (WT) strain UF-1 was cultured on potato dextrose agar at 25°C for 36 h before inoculation. A 5-mm inoculation disk taken from the growing margin of *S. sclerotiorum* was then patched onto leaves which were wrapped in bags containing wet gauze to maintain humidity. Inoculated plants were incubated at a constant temperature of 22°C in a greenhouse with 75–90% relative humidity. Leaf samples were collected at various time points. The samples were directly frozen in liquid nitrogen, freeze-dried, and stored at-80°C until RNA sequencing (RNA-seq) and quantitative reverse-transcription PCR (qRT-PCR).

### qRT-PCR

A first-strand cDNA synthesis kit (CWBIO, China) was used to reverse-transcribe 1 μg total RNA. The obtained cDNA was then diluted 10-fold and was used as a template for real-time PCR. qRT-PCR expression validation was performed, and specific primers were designed using the NCBI database (Supplementary Table S1). *GmEF-1α* and *AtActin 8* (AT1G49240) were used as housekeeping genes of soybean and *A. thaliana*, respectively. Reaction mixtures (10 μL) comprised DEPC-treated water and primers (0.4 μL), template cDNA (1 μL), and 2× TransStart Top Green qPCR SuperMix (5 μL; CWBIO, China). The thermocycling program, carried out on a Pro Real-Time System, consisted of 2 min at 50°C and 2 min at 95°C for pre-denaturation, followed by 44 cycles of 15 s at 95°C denaturation and extension for 20 s at 60°C and a melting curve. Reaction products were evaluated using the Pro Study software. Relative expression values were calculated using the 2^−ΔΔCT^ method, and the degrees of differential expression was expressed in terms of fold change (Ding et al., 2018).

### RNA extraction and sequencing

Total RNA was extracted from all samples using TriZol reagent. RNA integrity was assessed using the RNA Nano 6000 Assay Kit of the Bioanalyzer 2100 system (Agilent Technologies, CA, United States). The results were verified by agarose gel electrophoresis. Total RNA was used as input material for RNA sample preparation. Briefly, mRNA was purified from total RNA using poly-T oligo-attached magnetic beads. PCR was performed using Phusion High-Fidelity DNA polymerase, universal PCR primers, and an index (X) Primer. PCR products were purified (AMPure XP system), and library quality was assessed using an Agilent Bioanalyzer 2100 system. The clustering of the index-coded samples was performed on a cBot Cluster Generation System using TruSeq PE Cluster Kit v3-cBot-HS (Illumina, CA, United States) according to the manufacturer’s instructions. After cluster generation, the libraries were sequenced on an Illumina NovaSeq platform. Raw reads in fastq format were first processed using in-house Perl scripts. In this step, clean reads were obtained by removing reads containing adapters, ploy-N’s, and low-quality reads. At the same time, the Q20, Q30, and GC content of the clean data was calculated. All the downstream analyses were performed using high-quality clean data.

Glycine max strain Williams 82 RefSeq Genome (GCA_000004515.5 Glycine_max_v4.0, https://www.ncbi.nlm.nih.gov/genome/5?genome_assembly_id=1605618) and gene model annotation files were downloaded directly from the genome website. The index of the reference genome was produced using Hisat2 v2.0.5 and clean paired-end reads were aligned to the reference genome using Hisat2 v2.0.5.

Feature Counts v1.5.0-p3 was used to count the reads numbers mapped to each gene. Then, the FPKM of each gene was calculated based on the length of the gene and the number of reads mapped to the respective gene. The count data were normalized and log-transformed using the function calcNormFactors (trimmed mean of M-values normalization) of the R package edgeR and the function voomWithQualityWeights of the package limma, respectively.

### Differentially expressed gene identification and enrichment analyses

Differential expression analysis of the two conditions/groups (three biological replicates per condition) was performed using the R package DESeq2 (1.20.0). This program offers statistical pathways for determining differential expression in digital gene expression data using a model based on negative binomial distribution. The resulting *p-*values were adjusted using the Benjamini–Hochberg’s approach to control the false discovery rate. Differently expressed genes (DEGs) were identified using an adjusted *p*-value ≤ 0.05, and|log2[FoldChange]| ≥ 1 as the thresholds. Differential expression analysis in terms of the two conditions was performed using the R package edgeR (version 3.22.5).

Gene Ontology (GO) enrichment analysis of DEGs was performed using the R package clusterProfiler, during which gene length bias was corrected. GO terms with corrected *p*-values < 0.05 were considered significantly enriched with regard to DEGs. We used the R package clusterProfiler to test the statistical enrichment of DEGs in the Kyoto Encyclopedia of Genes and Genomes (KEGG) pathways, and a local version of the Gene Set Enrichment Analysis (GSEA) analysis tool^1^ was used, GO and KEGG datasets were independently used for GSEA.

### Protein–protein interaction network analyses

Protein–protein interaction (PPI) analysis of DEGs was executed using the on the STRING database, which contains known and predicted PPIs. Interactions with network data files were directly imported into Cytoscape software for visual editing. Each dot represents a protein, and the protein–protein interactions in the STRING protein interaction database are indicated by lines. When the number of interacting proteins of a given protein exceeded 20, this is indicated by larger circles, and different biological functions involved in the protein are represent by different colors.

### Weighted correlation network analysis and co-expression network analyses

Weighted correlation network analysis (WGCNA) is a systematic method to describe modes of gene associations among different samples. An adjacency matrix was constructed based on the normalized FPKM values. The R package for WGCNA was used for co-expression network analyses to evaluate modules in which genes showed high correlations. The adjacency matrix was further converted into a topological overlap matrix (TOM) using the WGCNA package. Transcripts with similar expression patterns were classified into one module (coefficient of dissimilarity < 0.25). The TOM and degree were subsequently calculated; the top 20 genes according to the degree were considered hub genes. Additionally, TOM > 0.3 indicated strong correlation and pronounced regulatory functions. The co-expression networks were visualized using Cytoscape software. Each dot represents a gene in the module and co-expression relationships are connected by lines. The network of the top hub genes is indicated by larger circles, and different biological functions involved in genes are represented by dots of different colors.

## Results

### Loss of *GmSWEET15* enhances soybean resistance to *Sclerotinia sclerotiorum*

**Figure 1:**
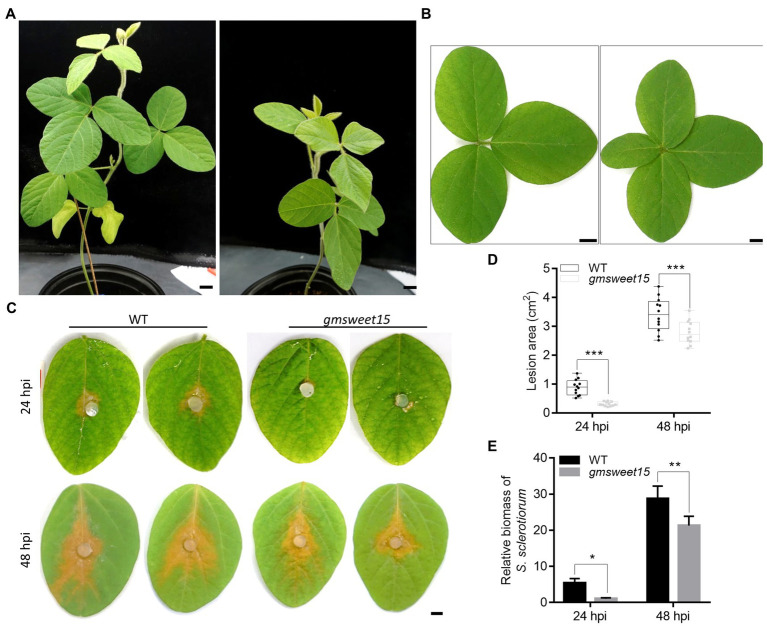
Loss of GmSWEET15 enhanced soybean resistance to *Sclerotinia sclerotiorum*. **(A)** Growth of WT and *gmsweet15-1* mutant plants after 6 weeks in the greenhouse. Scale bar = 2 cm. **(B)** Leaf morphology of WT and *gmsweet15-1* mutant second true leaves after 6 weeks. Scale bar = 1 cm. **(C)** Disease development on WT and *gmsweet15-1* mutant leaves after inoculation of *S. sclerotiorum* for 24 and 48 h. hpi hours post inoculation. Scale bar = 0.5 cm. **(D)** Area of lesion on WT and *gmsweet15-1* mutant leaves 24 and 48 hpi. **(E)** Relative biomass of *S. sclerotiorum* in WT and *gmsweet15-1* mutant leaves 24 and 48 hpi. Genomic DNA quantitative PCR, for which the *S. sclerotiorum β-tubulin* was amplified and *GmEF-1α* was used as a reference gene for normalizing soybean biomass. All above experiments were performed three times with similar results. Four plants were in each replicate of **A** and **B**, and 12 leaves in **C** and **D**. Asterisks indicate a statistically significant difference (^*^p < 0.05, ^**^p < 0.01, ^***^p < 0.001) according to a two-tailed comparisons *t*-test.

Previous studies have shown that GmSWEET15 is responsible for sucrose transport during seed development. However, the loss of *GmSWEET15* also delays senescence of the whole plant and yellowing of leaves during the vegetative growth stage (Wang et al., 2019b). In the present study, *gmsweet15* mutant plants and leaves were significantly smaller compared to WT plants after germination (Figures 1A, B), suggesting that GmSWEET15 may not exert only one isolated effect during seed development.

To explore whether GmSWEET15 affects the resistance of soybean to *S. sclerotiorum*, the lesion size of the WT and *gmsweet15-1* mutant inoculated with *S. sclerotiorum* was evaluated. The results showed that the lesion size of *gmsweet15-1* mutant was significantly smaller than that of the WT, at 24 h post inoculation (hpi) and at 48 hpi. Moreover, *gmsweet15-1* mutant largely blocked early colonization by *S. sclerotiorum* (Figures 1C, D). The relative *S. sclerotiorum* biomass was based on ratios of *S. sclerotiorum* DNA to soybean DNA *via* genomic DNA quantitative PCR was estimated. Compared with the WT, *gmsweet15-1* mutant exhibited a significantly decreased relative biomass of *S. sclerotiorum* (Figure 1E). These results suggested that *GmSWEET15* is associated with soybean susceptibility to *S. sclerotiorum*.

### Comparative transcriptome sequencing and qRT-PCR verification

**Figure 2:**
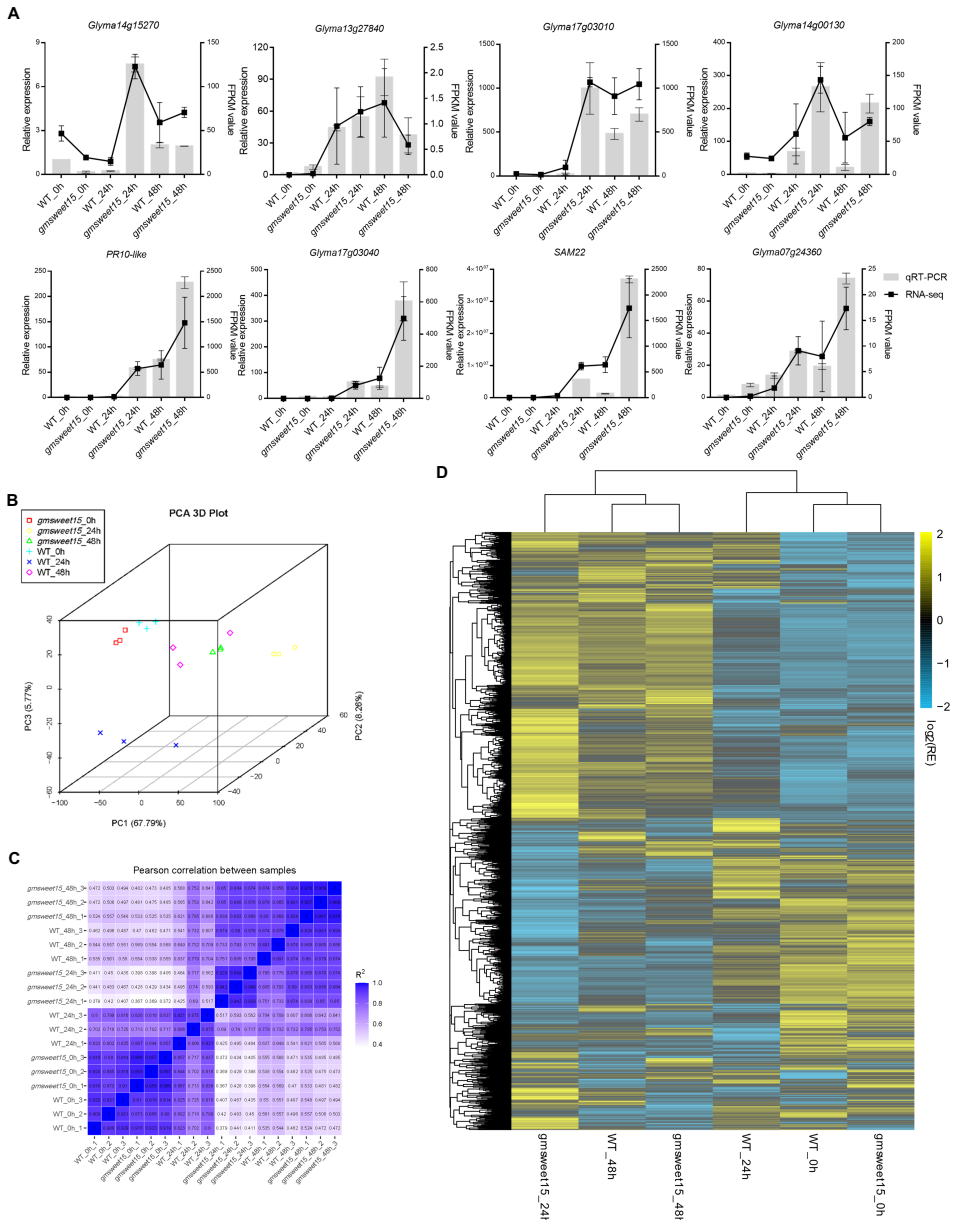
Assessment and full view of the RNA-seq. **(A)** qRT-PCR validation of eight randomly selected genes according to RNA-seq results. The qRT-PCR validation is shown as a bar chart, corresponding to the Y-axis on the left side, and *EF-1α* was used for each sample as an endogenous control. Relative expression values were determined against the average value of the WT_0h sample. The FPKM value was used as the expression level according to RNA-seq results, which is shown as a line on the right side. The standard deviation (*n* = 3) is represented by error bars. **(B)** Principal component analysis (PCA) of the transcriptome datasets. **(C)** Pearson correlation coefficient (R) of the transcriptome datasets. **(D)** Hierarchical clustering of relative expression (RE) of commonly detected all samples genes (in total, 21,461) in RNA-seq analysis. See Supplementary Tables S2A, B for gene expression data.

To further explore the mechanism of *gmsweet15* mutant resistance to *S. sclerotiorum*, comparative transcriptome sequencing was performed, including uninoculated WT and *gmsweet15* mutant (WT_0h and *gmsweet15*_0h), WT and *gmsweet15* mutant inoculated with *S. sclerotiorum* for 24 h (WT_24h and *gmsweet15*_24h), and WT and *gmsweet15* mutant inoculated with *S. sclerotiorum* for 48 h (WT_48h and *gmsweet15*_48h). Clean data comprising 113.33 Gb form 18 samples were obtained in each group of three replicates (Supplementary Figure S1). After alignment of the reference genome of soybean (GCA_000004515.5), the alignment rate of per sample was 83.55–96.73% (Supplementary Figure S1). To evaluate the reliability of RNA-seq data, we randomly selected eight genes for qRT-PCR verification. The results showed that the transcriptome sequencing was reliable in terms of overall trends and expression levels (Figure 2A). We then carried out PCA (Figure 2B) and tested Pearson’s correlation coefficient (R; Figure 2C) between samples to evaluate differences between groups and between sample replicates within groups. The results showed that R^2^ between biological replicates exceeded 0.8, whereas the samples between different groups showed different degrees of dispersion. Combined hierarchical clustering of relative expression of all commonly detected sample genes (21,461 in total) in the RNA-seq analyses (Figure 2D; Supplementary Tables S2A, B) showed that the expression patterns were significantly different after inoculation, indicating that a large number of genes exhibited transcription changes, especially *gmsweet15*_24h, which was significantly different from *gmsweet15*_0h, whereas WT was mainly different from *gmsweet15* mutant at 24 hpi, and there was little difference at uninoculated and 48 hpi, which suggested that the resistance phenotype of *gmsweet15* mutant may be mainly related to the dynamic transcriptional reprogramming response at the early stage of inoculation with *S. sclerotiorum*.

### Stronger and more widespread responses at the early stage of inoculation in the *gmsweet15* mutant

**Figure 3:**
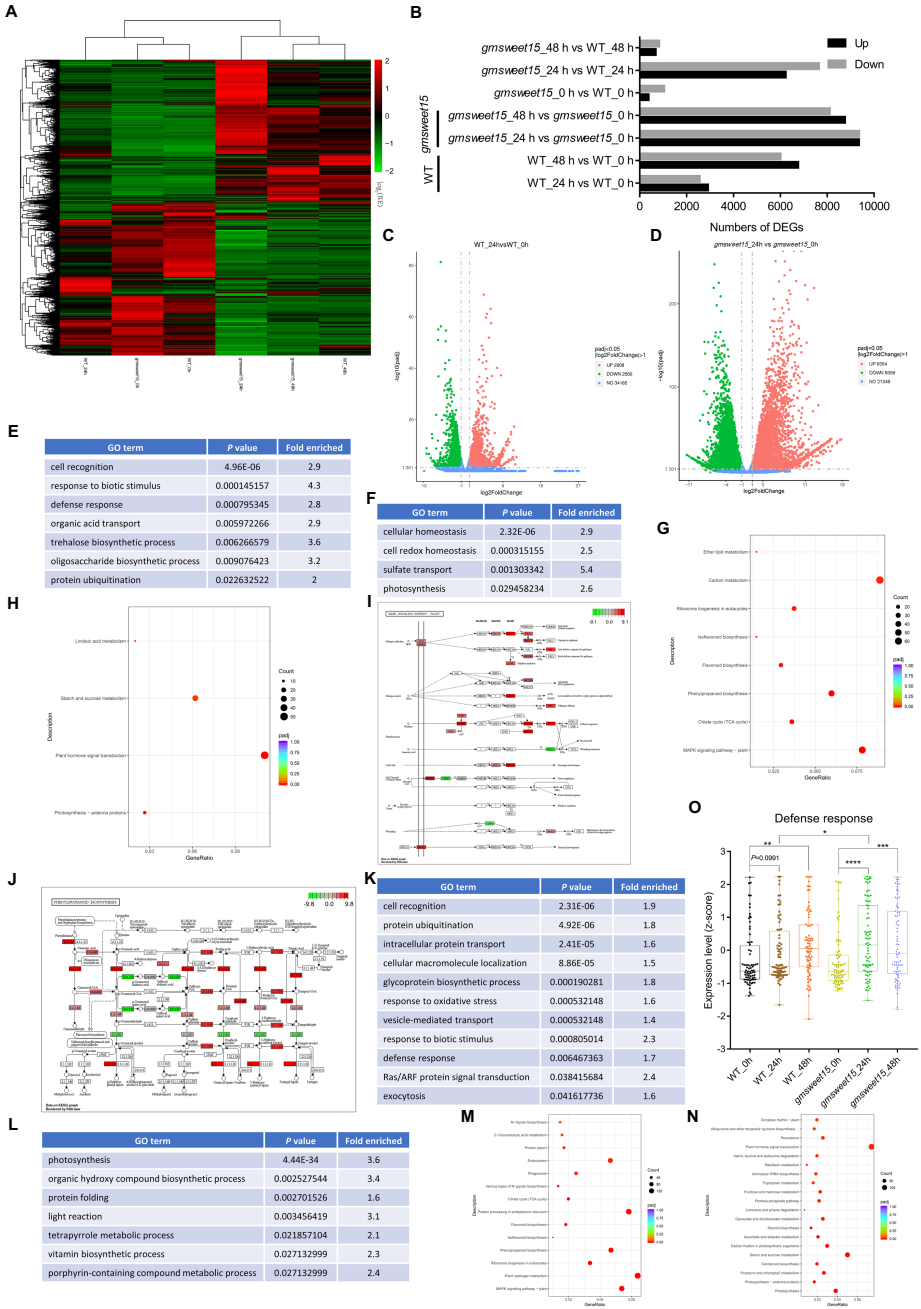
Pronounced transcription changes at the early stage of inoculation in gmsweet15 mutant. **(A)** Hierarchical clustering of the differentially expressed genes (DEGs) in RNA-seq. DEGs were identified using adjusted *p*-value ≤ 0.05 and |log_2_[FoldChange]| ≥ 1 as the threshold for the significance of the gene expression difference. See Supplementary Table S3 for gene expression data. **(B)** Statistical analysis of upregulated and downregulated DEGs in response to *S. sclerotiorum* infection and comparison between WT and *gmsweet15* mutant. **(C,D)** Volcano plot of the distribution of DEGs in response to (Continued)FIGURE 3 (Continued)*S. sclerotiorum* 24 hpi in WT **(C)** and *gmsweet15* mutant **(D)**. The X-axis represents the change in gene expression in different samples, here represented by log_2_[FoldChange] value. The Y-axis indicates the statistical significance of changes in gene expression levels, here represented by-log10 (padj) value. The dots represent different genes. The blue dots represent genes with no significant difference, the red dots represent genes with significant differences in upregulation, and the green dots represent genes with significant differences in downregulation. **(E,F)** Significantly enriched gene ontology (GO) biological process terms in the upregulated **(E)** and downregulated **(F)** DEGs in WT_24 h vs. WT_0 h. **(G,H)** Significantly enriched Kyoto Encyclopedia of Genes and Genomes (KEGG) pathways in the upregulated **(G)** and downregulated **(H)** DEGs in WT_24 h vs. WT_0 h. **(I,J)** DEGs of significantly enriched MAPK signaling pathway-plant pathway **(I)** and phenylpropanoid biosynthesis pathway **(J)** in WT_24 h vs. WT_0 h. The ruler is the largest log_2_[FoldChange] value of all the differentially annotated genes into this pathway. Red indicates that the genes annotated to the pathway are upregulated, and green indicates that the genes annotated to the pathway are downregulated. **(K,L)** Significantly enriched GO biological process terms in the upregulated **(K)** and downregulated **(L)** DEGs in *gmsweet15*_24 h vs. *gmsweet15*_0 h. **(M,N)** Significantly enriched KEGG pathways in the upregulated **(M)** and downregulated **(N)** DEGs in *gmsweet15*_24 h vs. *gmsweet15*_0 h. **(O)** Box plots showing expression level (z-score) related to defense response in global gene. Box plots with boxes displaying the 25th–75th percentiles, the center line indicating the median. All individual data points are overlaid. Asterisks indicate a statistically significant difference (*p < 0.05, **p < 0.01, ***p < 0.001, ****p < 0.0001) according to a two-tailed comparisons *t*-test. In E-N, the adjusted *p*-value ≤ 0.05 is used as the threshold of significant enrichment in GO and KEGG enrichment. For expression data and full GO or KEGG lists, see Supplementary Tables S4–S7.

To explore which genes were affected by *GmSWEET15* under inoculation with *S. sclerotiorum*, we analyzed the DEGs. A total of 24,993 DEGs were identified, and cluster heat maps were generated according to our screening criteria (Figures 3A, B; Supplementary Table S3). We found that 2,908 genes and 2,560 genes were upregulated and downregulated, respectively, in WT_24 h vs. WT_0 h (Figures 3B, C; Supplementary Tables S4A, B). While *gmsweet15*_24 h vs. *gmsweet15*_0 h showed stronger differential expression patterns, with 9,354 genes and 9,356 genes were upregulated and downregulated, respectively (Figures 3B, D; Supplementary Tables S4C, D). Comparing the gene expression levels at 48 and 0 hpi, 12,755 and 16,851 DEGs were identified in WT and *gmsweet15* mutant, respectively (Figure 3B; Supplementary Table S5). Above all, when comparing the gene expression levels in WT and *gmsweet15* mutant, only 1,405 and 1,501 DEGs were identified at uninoculated and 48 hpi, respectively, whereas 13,862 DEGs were observed at 24 hpi (Figure 3B; Supplementary Table S6). This suggests that *gmsweet15* mutant exhibited stronger transcriptional changes in the early stage of inoculation than the WT, which was consistent with the analysis of the relative expression relationships of the samples.

GO and KEGG analyses were carried out to determine which biological processes and pathways were enriched in terms of the DEGs. Among the upregulated DEGs of WT_24 h vs. WT_0 h, cell recognition, response to biotic stimulus, and defense response, among others, were identified by GO enrichment analysis (Figure 3E; Supplementary Table S7A). Biological processes, such as cell redox homeostasis and photosynthesis were significantly downregulated (Figure 3F; Supplementary Table S7B). In addition, pathways such as MAPK signaling pathway-plant and phenylpropanoid biosynthesis were significantly upregulated, whereas photosynthesis-antenna proteins and plant hormone signal transduction were significantly downregulated (Figures 3G, H; Supplementary Tables S7C, D). Here, several genes of MAPK cascades were significantly upregulated (Figure 3I; Supplementary Table S7C), such as upstream receptor *BAK1*, intermediate cascade kinases *MKK2* and *MPK4*, substrate *MKS1*, *ACS6*, and downstream transcription factors including *WRKY29*, *WRKY33*, and *EIN3*. Additionally, phenylpropanoid biosynthesis pathway had also undergone significant reprogramming (Figure 3J; Supplementary Table S7C), which included enzyme genes such as phenylalanine ammonia-lyase (*PAL*), trans-cinnamate 4-monooxygenase (*C4H*), cinnamoyl-CoA reductase (*CHR*), caffeoyl-CoA O-methyltransferase (*CCoMT*), lignin synthetases, cinnamyl alcohol dehydrogenase (*CAD*) and chalcone synthase (*CHS*). These results indicated that inoculation with *S. sclerotiorum* activated a certain number of defense-related signaling pathways, and the synthesis of secondary metabolites, but inhibited photosynthesis.

In contrast, *gmsweet15* mutant showed more extensive and intense transcriptional reprogramming in the early stage of inoculation. Two major findings were produced. The first is that more specific biological processes were enriched in *gmsweet15*_24 h vs. *gmsweet15*_0 h, including intracellular protein transport, glycoprotein biosynthetic process, response to oxidative stress, Ras/ARF protein signal transduction, exocytosis, plant-pathogen interaction, protein processing in endoplasmic reticulum, and endocytosis were up-expressed (Figures 3K, M; Supplementary Tables S7E, G). Protein folding, tetrapyrrole metabolic process, steroid biosynthesis, and peroxisome processes were downregulated (Figures 3L, N; Supplementary Tables S7F, H). Secretory pathways such as endoplasmic reticulum processing, exocytosis, and protein transport were specifically upregulated, which implied that *gmsweet15* mutant may have organized more abundant extracellular peptides such as PR1 as a weapon oppose primary infection of *S. sclerotiorum*, which was consistent with *gmsweet15*_24 h exhibiting higher *PR1* expression than WT_24 h (Supplementary Table S3). The downregulation of some tetrapyrrole biosynthesis enzymes may lead to the accumulation of photosensitive intermediates in leaves and to cell death (Ishikawa et al., 2001; Chai et al., 2017). Such as coproporphyrinogen-III oxidase (*CPX*), was downregulated 3.4-fold (Supplementary Table S3), which may enhance immunity by reducing the biosynthesis of tetrapyrrole, as knock-out of *GmCPX* can significantly enhance the resistance of soybean to *Phytophthora sojae* (Ma et al., 2020). These results suggest that *gmsweet15* mutant exhibited enrichment of more biological processes than WT in the early stage of inoculation with *S. sclerotiorum*, and the rearrangement of these pathways may play an important role in delaying infection with *S. sclerotiorum* in *gmsweet15* mutant.

A significantly stronger upregulation of immunity and downregulation of photosynthesis occurred *gmsweet15* mutant, although these processes were also enriched in WT after inoculation (Figure 3; Supplementary Table S7). To elucidate the biological processes or pathways that were differentially regulated under various conditions, all genes associated with a respective term in our transcriptome were selected, and standardized expression scores were calculated. The GO term defense response showed pronounced upregulation in *gmsweet15* mutant after inoculation, especially at 24 hpi, while significant upregulation in WT occurred only at 48 hpi. Moreover, the expression was higher in the *gmsweet15*_24 h group than in the WT_24 h group (Figure 3O). This indicates that *gmsweet15* mutant had a more powerful arsenal by upregulating the genes of defense response after sensing *S. sclerotiorum*, which may directly lead to *gmsweet15* mutant gaining disease resistance.

### Identification and classification of the DEGs between *gmsweet15* mutant and WT

To further explore the key genes and biological processes involved in *gmsweet15* mutant resistance to *S. sclerotiorum*, we deployed the differential expression analysis of WT and *gmsweet15* mutant before and after inoculation for the same time. Compared to the WT, 369 genes were upregulated and 1,036 genes were downregulated in the *gmsweet15* mutant without inoculation (Figure 3B; Supplementary Tables S6A, B). In this case, GO terms such as polysaccharide catabolic process and response to chemical and drug transmembrane transport were upregulated, whereas protein ubiquitination and aminoglycan metabolic processes were downregulated (Supplementary Figure S2A; Supplementary Table S8A). In addition, terms such as MAPK signaling pathway-plant, plant-pathogen interaction, and plant hormone signal transduction were enriched (Supplementary Figures S2B, C; Supplementary Table S8B). Interestingly, the expression levels of genes such as *MPK3*, *RBOHB*, *Pti1*, *SGT1* which have long been considered to contribute to disease resistance (Forzani et al., 2011; Hawamda et al., 2020; Yu G. et al., 2020; Zhang and Zhang, 2022), were lower in *gmsweet15* mutant than in WT (Supplementary Figure S2D; Supplementary Table S6B). Considering that there were not many DEGs and no obvious functional enrichment in accordance with the basis of resistance, we propose that the difference in uninoculated is not the reason for the resistance of *gmsweet15* mutant to *S. sclerotiorum*.

**Figure 4:**
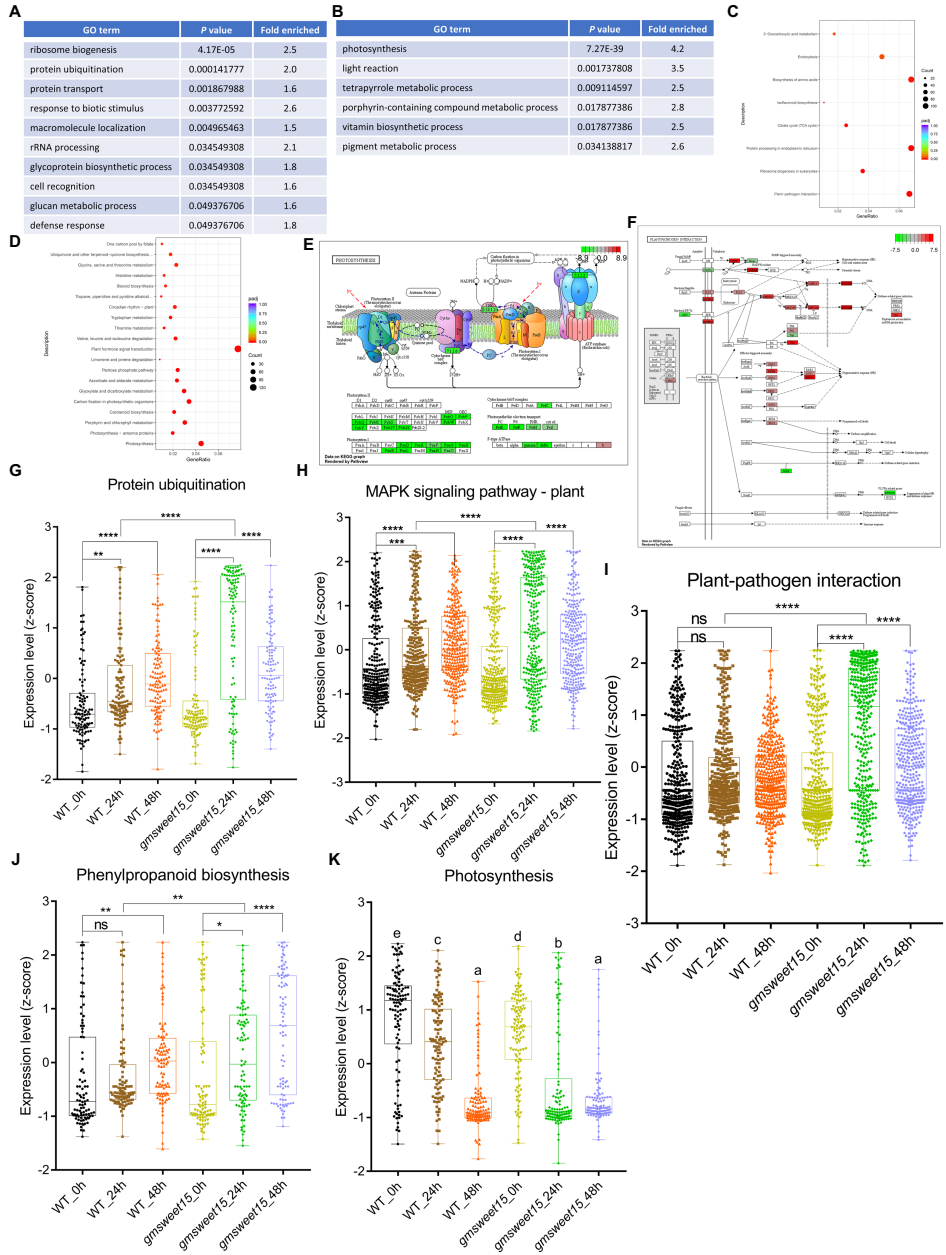
Characterization of the DEGs between *gmsweet15* mutant and WT. **(A,B)** Significantly enriched GO biological process terms in the upregulated **(A)** and downregulated **(B)** DEGs in *gmsweet15*_24 h vs. WT_24 h. **(C,D)** Significantly enriched KEGG pathways in the upregulated **(C)** and downregulated **(D)** DEGs in *gmsweet15*_24 h vs. WT_24 h. (E, F) DEGs of significantly enriched photosynthesis pathway **(E)** and plant-pathogen interaction pathway **(F)** in *gmsweet15*_24 h vs. WT_24 h. **(G–K)** Box plots showing expression level (z-score) related to protein ubiquitination **(G)**, MAPK signaling pathway-plant **(H)**, plant-pathogen interaction **(I)**, phenylpropanoid biosynthesis **(J)** and photosynthesis **(K)** in global gene. Asterisks indicate a statistically significant difference (*p < 0.05, **p < 0.01, ***p < 0.001, ****p < 0.0001) according to a two-tailed comparisons *t*-test, and ns represent no statistical difference. For **K**, different letters indicate statistically significant differences (adjusted *p* < 0.05, two-tailed Student’s *t*-test, Benjamini–Hochberg method). For expression data and full GO or KEGG lists, see Supplementary Table S8.

In contrast, a wealth of DEGs was identified in *gmsweet15*_24 h vs. WT_24 h, including 6,223 upregulated and 7,639 downregulated genes (Figure 3B; Supplementary Tables S6C, D). Functional analysis of these DEGs revealed that the enriched categories were almost the same as those enriched by *gmsweet15*_24 h vs. *gmsweet15*_0 h. They all were similarly upregulated in biological processes and pathways such as protein ubiquitination, protein transport, defense response, glycoprotein biosynthetic, plant-pathogen interaction, protein processing in endoplasmic reticulum, ribosome biogenesis, and MAPK signaling pathway-plant. Photosynthesis and tetrapyrrole metabolic processes were downregulated (Figures 4A–D; Supplementary Tables S8C–F). Notably, abundant genes in ribosome biosynthesis were significantly upregulated, including nucleolar protein 56 (*NOP56*), small nuclear ribonucleoprotein 13 (*SNU13*), and H/ACA ribonucleoprotein complex subunit 2 (*NHP2*) responsible for rRNA modification, U3 small nucleolar RNA-associated protein 14 (*UTP14*), nucleolar GTP-binding protein (*Nog1*), and 60S ribosomal export protein (*NMD3*) responsible for pre-ribosome cleavage and transport and almost all components of the 90s pre-ribosome precursor (Figures 4A, C; Supplementary Table S8E).

Inhibition of photosynthesis is frequently observed in plants under various biotic stresses. Compared with the WT, *gmsweet15* mutant showed more obvious photoinhibition, and all components of the photosynthetic apparatus were downregulated to different degrees, among which *PsbO*, *PsbP* and *PsbQ* of photosystem II oxygen-evolving enhancer protein were significantly downregulated (Figure 4E; Supplementary Table S8F). Furthermore, compared with the WT, *gmsweet15* mutant also comprehensively enhanced the plant-pathogen interaction pathway, including membrane recognition receptors such as *BAK1*, chitin elicitor receptor kinase 1 (*CERK1*) and flagellin sensitive 2 (*FLS2*), various components of PTI, such as cyclic nucleotide gated channel (*CNGCs*), calcium-dependent protein kinase (*CDPK*), *RBOH*, calmodulin (*CaM*), *MAPKs*, *WRKY33* and *PR1*, or some key nodes of ETI such as resistance to *Pseudomonas maculicola* 1 (*RPM1*), RPM1-interacting protein 4 (*RIN4*), AvrPphB susceptible 1 (*PBS1*), *SGT1*, heat shock protein 90 (*HSP90*), and enhanced disease susceptibility 1 (*EDS1*; Figure 4F; Supplementary Table S8E). The upregulated expression of these defense genes may largely explain why the *gmsweet15* mutant was able to delay colonization by *S. sclerotiorum*.

Moreover, consistent with the GO enrichment analysis, the global gene of protein ubiquitination of *gmsweet15* mutant was significantly upregulated, which was more pronounced than in the WT (Figure 4G). In addition, at the early stage of inoculation with *S. sclerotiorum*, the global genes of MAPK signaling pathway-plant, plant-pathogen interaction, and phenylpropanoid biosynthesis were markedly upregulated in *gmsweet15* mutant (Figures 4H–J), while photosynthesis showed more significant downregulation (Figure 4K). These data are consistent with the previous analyses of DEGs (Figures 3, 4). In general, the more intense and abundant transcriptional changes in *gmsweet15* mutant may explain why it was able to effectively delay colonization by *S. sclerotiorum*.

### Venn diagram showing common and specific DEGs between WT and *gmsweet15* mutant

**Figure 5:**
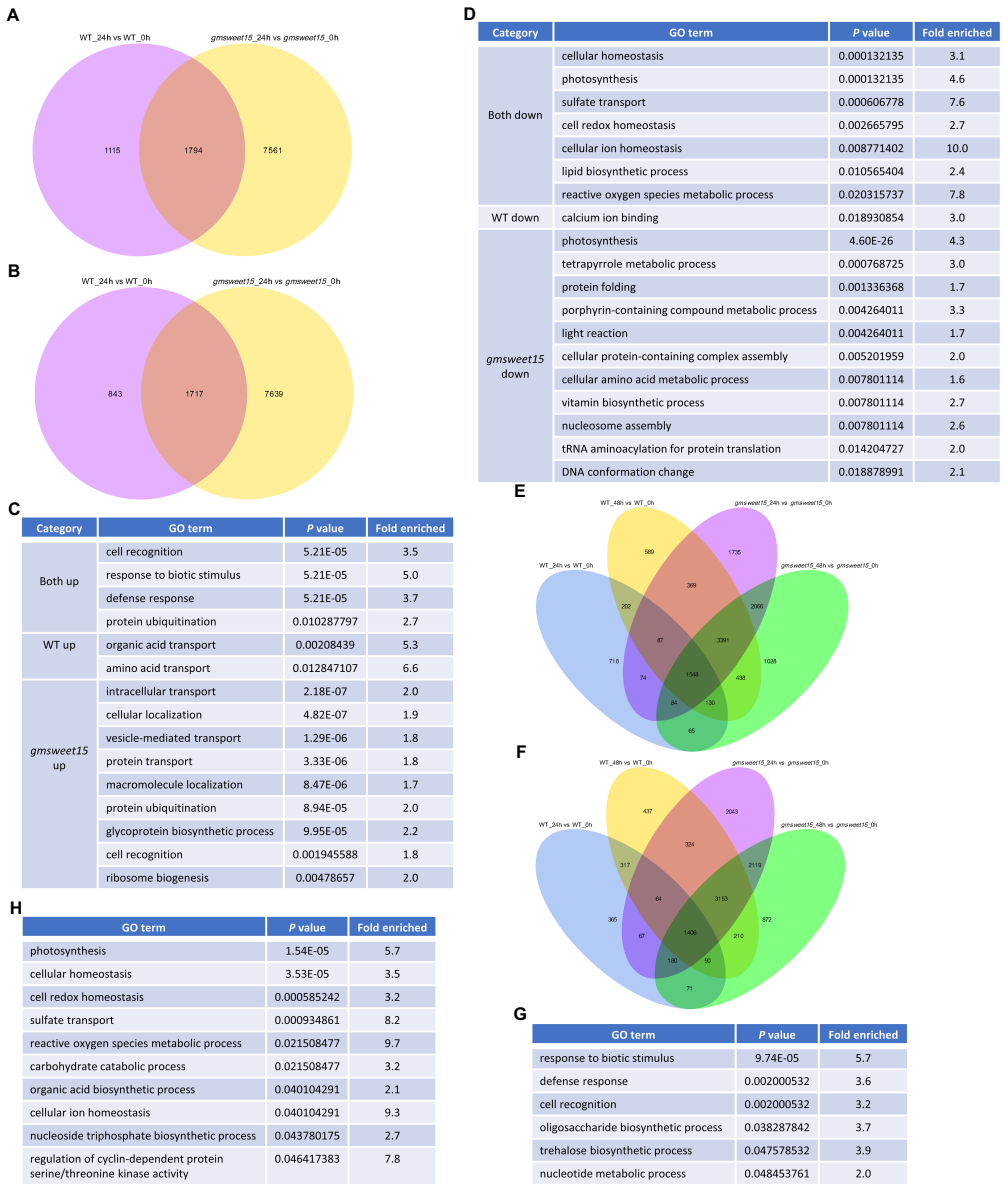
Characterization of common and specific DEGs between WT and *gmsweet15* mutant. **(A,B)** Venn diagram showing the overlaps and specific upregulated **(A)** or downregulated **(B)** DEGs in WT and *gmsweet15* mutant at 24 hpi. **(C,D)** Significantly enriched GO biological process terms in the upregulated **(C)** and downregulated **(D)** common and specific DEGs in WT and *gmsweet15* mutant at 24 hpi. **(E,F)** Venn diagram showing the overlaps and specific upregulated **(E)** or downregulated **(F)** DEGs in WT and *gmsweet15* mutant at 24 and 48 hpi. **(G,H)** Significantly enriched GO biological process terms in the upregulated **(G)** and downregulated **(H)** common and specific DEGs in WT and *gmsweet15* mutant at 24 and 48 hpi. For expression data and full GO lists, see Supplementary Table S9.

To explore the unique and common response mechanism of WT and *gmsweet15* mutant after inoculation with *S. sclerotiorum*, we indicated the common and unique DEGs between WT and *gmsweet15* mutant after inoculation in a Venn diagram. Under the condition of upregulation at 24 hpi, 1,794 common DEGs between WT and *gmsweet15* mutant were identified, while 1,115 and 7,561 unique DEGs were identified in the WT and *gmsweet15* mutant, respectively (Figure 5A; Supplementary Table S9A). In the context of downregulation at 24 hpi, 1,717 common DEGs were obtained, whereas 843 and 7,639 unique DEGs in WT and *gmsweet15* mutant were identified, respectively (Figure 5B; Supplementary Table S9B). GO enrichment analysis of these upregulated DEGs showed that response to biotic stimulus and defense response terms were common biological processes, amino acid transport and organic acid transport were only upregulated in WT, while several secretion pathways and glycoprotein biosynthetic process were specifically upregulated in *gmsweet15* mutant (Figure 5C; Supplementary Table S9C). With regard to the downregulated DEGs, cellular homeostasis and photosynthesis were common to both, and the WT specifically showed downregulated calcium ion binding terms, while the mutant not only showed downregulated another part of photosynthesis genes, but also inhibited the tetrapyrrole metabolic process (Figure 5D; Supplementary Table S9D).

Among the DEGs altered by *S. sclerotiorum* infection, 12,524 genes were induced, of which 1,548 genes were upregulated under all conditions (Figure 5E; Supplementary Table S9E). In contrast, 11,718 genes were inhibited and 1,406 genes were downregulated under all conditions (Figure 5F; Supplementary Table S9F). Among the 1,548 genes, defense response and trehalose biosynthetic process were enriched (Figure 5G; Supplementary Table S9G). Among the genes that were downregulated together, terms such as photosynthesis, cellular homeostasis, and reactive oxygen species metabolic process were enriched (Figure 5H; Supplementary Table S9H). Collectively, Venn diagrams identified common and specific DEGs between WT and *gmsweet15* mutant after inoculation with *S. sclerotiorum*.

### GSEA identified gene sets related to *gmsweet15* mutant

GSEA is frequently used to identify gene sets that are closely related to particular phenotypes. To explore which gene sets were related to early disease resistance of *gmsweet15* mutant, we performed GSEA on two comparative combinations, i.e., *gmsweet15*_24 h vs. *gmsweet15*_0 h and *gmsweet15*_24 h vs. WT_24 h. In the former, 308 and 262 gene sets were significantly upregulated in *gmsweet15*_24h and *gmsweet15*_0 h, respectively (Supplementary Tables S10A, B). In the latter, 96 and 135 gene sets were significantly upregulated in *gmsweet15*_24 h and WT_24 h, respectively (Supplementary Tables S10C, D).

Selecting the top 20 gene sets with the highest enrichment degree for display, it can be seen that in addition to the gene sets of defense type described above, glucose metabolic process, glycosylation, calmodulin binding, phosphotransferases, ligand gated ion channel activity, and plasma membrane protein complex were upregulated in *gmsweet15*_24 h (Supplementary Figure S3A; Supplementary Table S10A). Correspondingly, the gene sets for interconverting aldoses and ketoses, heat shock protein binding, and DNA conformation change were enriched in *gmsweet15*_0 h (Supplementary Figure S3B; Supplementary Table S10B). In the comparative combination of *gmsweet15*_24 h and WT_24 h, *gmsweet15*_24 h was additionally enriched with vesicle mediated transport, organelle membrane and endoplasmic reticulum, although the enriched gene set of WT_24 h was similar that of *gmsweet15*_0 h (Supplementary Figures S3D, E; SupplementaryTables S10C, D).

Additionally, GSEA identified some genes that were strongly associated with the corresponding phenotypes in the two groups of comparative combinations. Genes such as terpene synthase 14 (*TPS14*), heat stress transcription factor (*HSF5*, *HSF36*), wound-induced protein (*WIN*), *PR-10*, and cysteine rich receptor-like protein kinase (*CRK79*, *CRK63*), heat shock protein 90-A1 (*HSP90A1*), cytochrome P450 82A2-like (*CYP82A2*), were positively correlated with the phenotype of *gmsweet15*_24 h, whereas the bZIP transcription factor (*BZIP19*), lipoxygenase-9 (*LOX9*), auxin transporter-like protein 12 (*LAX12*), CONSTANS-like 2a (*COL2A*), ammonium transporter *AMT1.4*, MYB transcription factor *MYB164* and CBL-interacting protein kinase 23 were negatively correlated (Supplementary Figures S3C, F; Supplementary Tables S10E, F). Identification of these genes provides a promising breeding resource for to SSR resistance. Collectively, GSEA identified gene sets and genes related to *gmsweet15*_24 h.

### Constructing PPI network to identify candidate core proteins

First, we applied the interaction relationship in the STRING protein interaction database to construct a PPI network of the DEGs. Then, according to KEGG annotation and the number of interacting proteins, each protein was assigned a color and node size (Supplementary Figure S4). Proteins in the same pathway showed distinct aggregation, which suggested that genes not annotated by KEGG in a certain pathway may have similar functions. For example, GLYMA_04G101000 (referred to as PPP1) encodes a putative protein, although predicted to contain a domain of protein phosphatase 1 regulatory subunit 42, clustered in the plant hormone signal transduction pathway (Supplementary Figure S4). This suggested that it may also be involved in hormone signal transduction. Moreover, the PPI network showed that PPP1 interacted with GLYMA_04G147000 (EIN3-binding F-box protein 1, EBF1) and GLYMA_16G064100 (MDIS1-interacting receptor like kinase 2, MIK2), respectively, and MIK2 also interacted with EBF1 (Potuschak et al., 2003; Van der Does et al., 2017; Hou et al., 2021). These results imply that PPP1 may act as a phosphatase to fine-tune the ethylene response through dephosphorylation of EBF1 and phosphorylation of EBF1 by MIK2.

In addition, we have also identified some core proteins through the PPI network, and here, those with more than 20 interactions were designated core proteins. GLYMA_11G010500 (4-coumarate: CoA ligase isoenzyme 3, 4CL3) interacted with 27 proteins, most of which were involved in phenylpropanoid biosynthesis (Supplementary Figure S4), which emphasizes the important role of 4CL3 in phenylpropanoid biosynthesis. Collectively, we constructed a rich PPI network that was helpful for the discovery of candidate functional genes.

### Gene co-expression analysis predicts logic of regulatory response to *Sclerotinia sclerotiorum* in *gmsweet15* mutant

**Figure 6:**
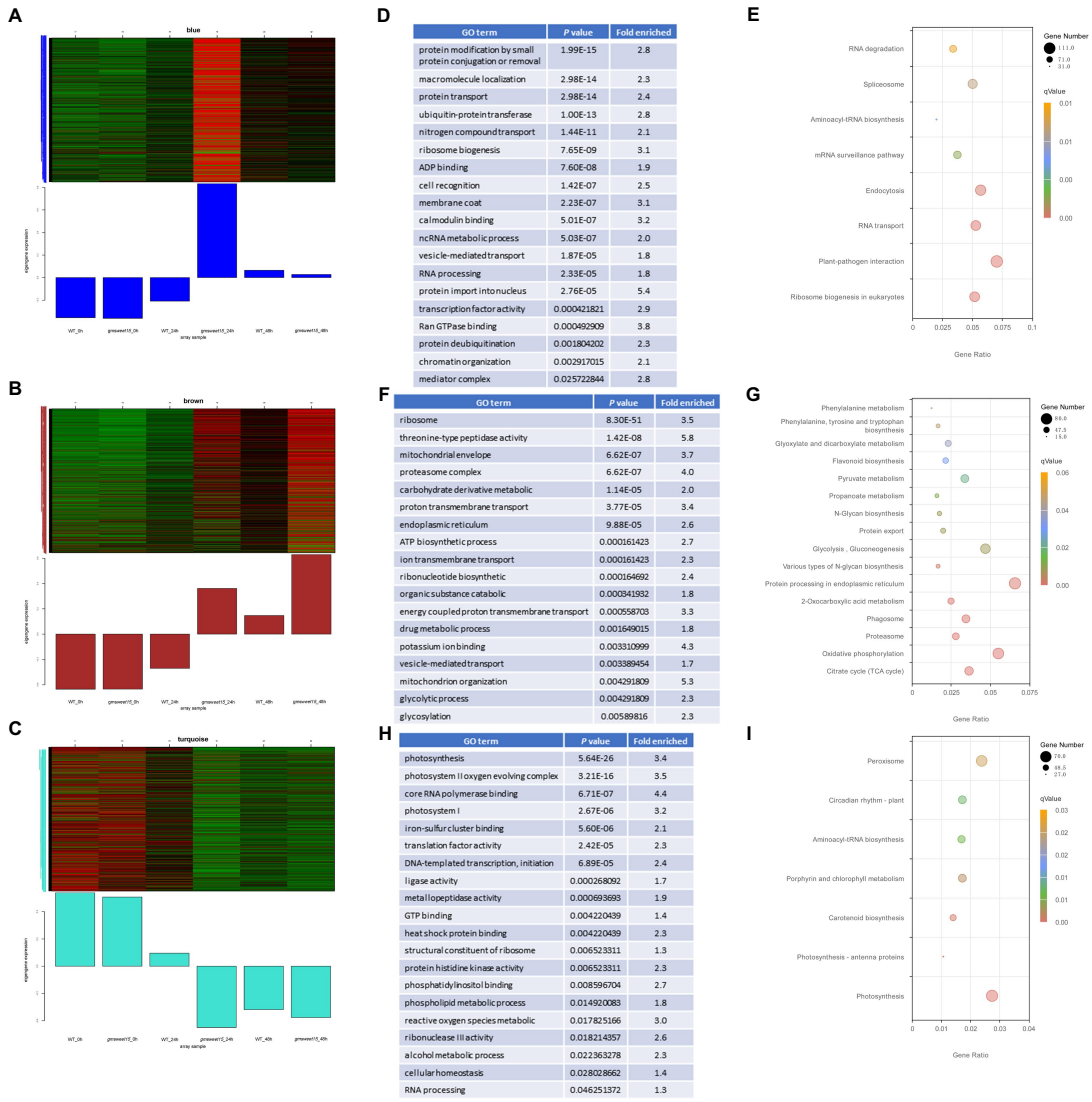
Characterization of gene modules associated with gmsweet15 mutant. **(A–C)** The co-expressed genes are shown in heatmaps and bar graphs in the blue module **(A)**, brown module **(B)** and turquoise module **(C)**. The red rectangles in the heatmap represent high expression; the green rectangles represent low expression. **(D,E)** Significantly enriched GO terms **(D)** or KEGG pathways **(E)** of the blue module. **(F,G)** Significantly enriched GO terms **(F)** or KEGG pathways **(G)** of the brown module. **(H,I)** Significantly enriched GO terms **(H)** or KEGG pathways **(I)** of the turquoise module. For expression data and full GO or KEGG lists, see Supplementary Table S11.

To deconvolute the gene regulatory network of the response to *S. sclerotiorum* in *gmsweet15* mutant, we performed WGCNA on 3,2017 genes with expression levels. A total of 30 highly correlated modules were obtained (Supplementary Figures S5A–C). Distinct positive correlations between several modules and samples were observed (Supplementary Figure S5D). Notably, the blue module showed a specific high expression in *gmsweet15*_24 h, the brown module showed a trend of upregulation after inoculation, but *gmsweet15* mutant exhibited a more substantial upregulation, while the turquoise module showed a downward trend after inoculation, contrary to the brown module, and the downregulation in *gmsweet15* mutant was more pronounced (Figures 6A–C; Supplementary Tables S11A–C).

Functional enrichment analysis was performed for the three modules. The blue module was enriched with respect to terms including plant-pathogen interaction, ubiquitination modification, protein transport, ribosome biosynthesis, calcium signaling, and mediator complex (Figures 6D, E; Supplementary Table S11D). The enrichment of these terms in the blue module further confirmed that genes associated with this module are an important in the defense of *gmsweet15* mutant during early *S. sclerotiorum* infection. In the brown module, terms such as proteasome, ATP biosynthetic process, glycosylation, endoplasmic reticulum, and flavonoid biosynthesis were enriched (Figures 6F, G; Supplementary Table S11E). Although the turquois module, terms such as photosynthesis, metallopeptidase activity, heat shock protein binding, phosphatidylinositol binding, ROS metabolism, circadian rhythm, and peroxisome were enriched (Figures 6H, I; Supplementary Table S11F).

**Figure 7:**
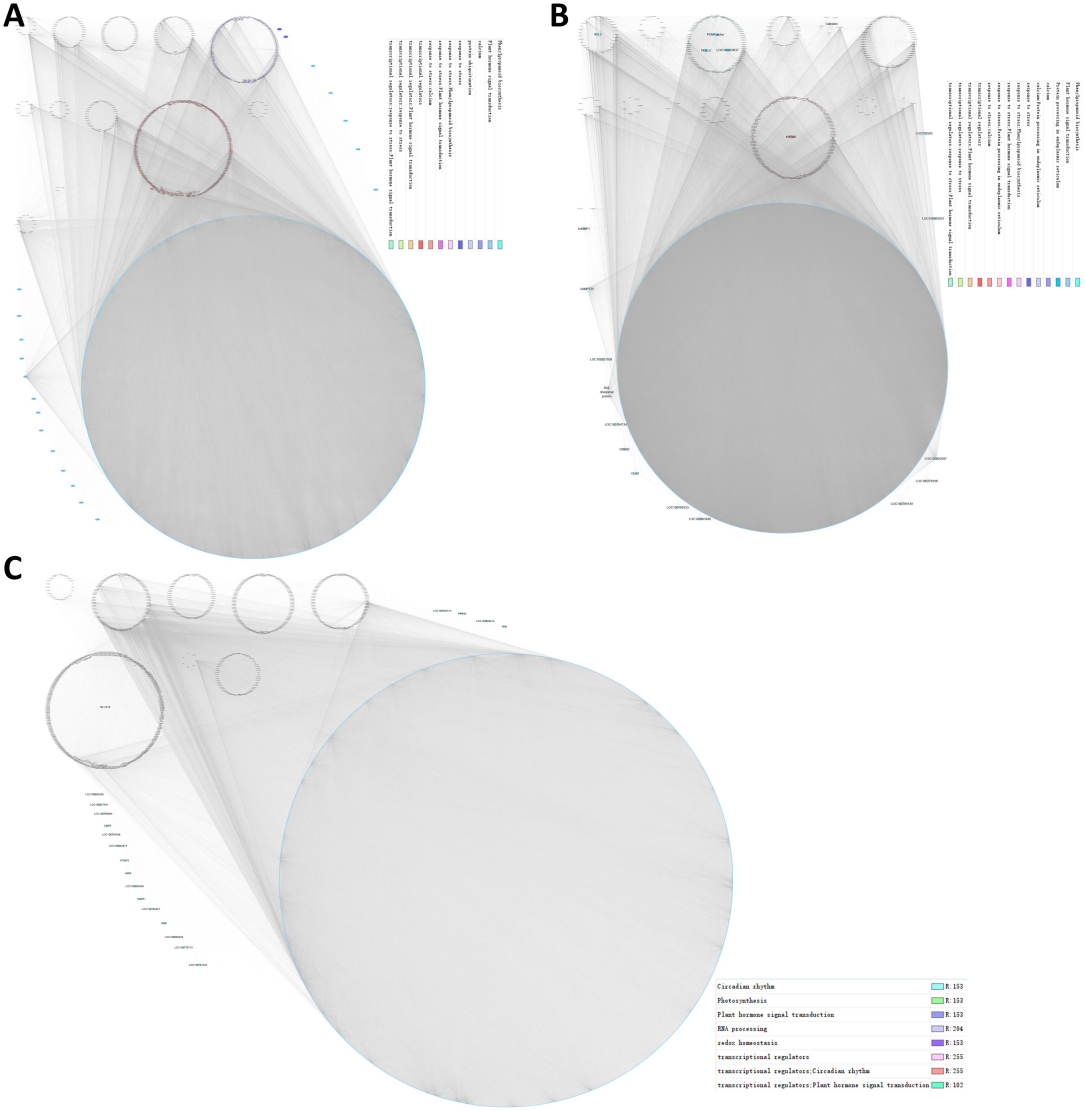
Regulatory networks of *gmsweet15* mutant response to *S. sclerotiorum* infection. **(A–C)** Co-expression network of the blue module **(A)**, brown module **(B)**, and turquoise module **(C)**. Each module was visualized using Cytoscape. Each dot represents a gene in the module, and the co-expression relationships are connected by lines. The network of top hub genes is indicated by larger circles and different biological functions involved in genes are represented by dots of different colors.

Then, a co-expression network was constructed according to the correlation strength of the genes in each module, and we identified hub genes in each module where several biological processes clustered together (Figure 7). Notably, in the blue module, two stress-responsive genes, i.e., septum-promoting GTP-binding protein 1 (*spg1*; Guan and Nothnagel, 2004; Rizhsky et al., 2004; Sottosanto et al., 2007; Ascencio-Ibanez et al., 2008) and Pti1 kinase-like protein (*PTI1B*; Forzani et al., 2011), were considered hub genes (Figure 7A). In the brown module, MYB86 transcription factors, calnexin, 4CL3, and the three endoplasmic reticulum proteins GTP-binding protein SAR1A, translocon-associated protein subunit alpha (TRAP-alpha), and protein disulfide isomerase-like protein (PDIL-2) were identified as hub genes (Figure 7B). In addition, the NF-YA15 transcription factor was a hub gene in the turquois module (Figure 7C). We anticipated that transcription factors in hub genes should be highly co-expressed in a group with its target. Here, *MYB86* was highly co-expressed with 268 genes, including 8 other transcription factors, 12 genes response to stress, 11 genes of phenylpropanoid biosynthesis, and 3 other hub genes, indicating that MYB86 may regulate the above biological processes and the expression of the respective genes. NF-YA15 may regulate the expression of 63 genes, including 16 photosynthesis genes, 2 redox homeostasis genes, and the transcription factor GLYMA_16G017400 (late elongated hypocotyl and circadian clock associated-1-like protein 1, *LCL1*) of the circadian rhythm. Collectively, we identified several groups of regulatory networks of *gmsweet15* mutant in response to *S. sclerotiorum* infection using co-expression network analysis.

## Discussion

Starvation-induced resistance strategies using *SWEETs* as susceptibility genes have been applied in disease-resistant rice breeding (Li et al., 2012; Oliva and Quibod, 2017; Oliva et al., 2019). However, with regard to the interactions of soybean and *S. sclerotiorum*, the role of SWEET and potential molecular processes are unclear. In the current study, loss of *GmSWEET15* enhanced soybean resistance to *S. sclerotiorum*, and a series of biological processes and functional genes that may play a key role in the response of *gmsweet15* mutant response to *S. sclerotiorum* infection were identified by comparative transcriptome profiling. It was preliminarily determined that increased dynamic transcriptional reprogramming in the early stage of inoculation was an important reason for *gmsweet15* mutant being able to decelerate colonization by *S. sclerotiorum*. Our research elucidated the molecular mechanism by which *gmsweet15* mutant were able to increase their resistance to *S. sclerotiorum*, and we propose numerous candidate functional genes for elevating resistance to SSR.

*SWEETs* encode for sugar transporters, which are usually considered as susceptibility genes, while their recessive alleles provide resistance (Gao et al., 2018; Gupta, 2020; Xue et al., 2022). TALE effectors secreted by pathogenic bacteria induce the expression of *SWEETs* in the host and promote the release of more sugars into the apoplast, which are then consumed by pathogenic bacteria (Kay et al., 2009; Cox et al., 2017; Gupta et al., 2021). Unlike pathogenic bacteria, no similar TALE effectors have been identified in plant pathogenic fungi, although numerous studies reported that pathogenic fungi induce expression of *SWEETs* in the host (Miao et al., 2017; Li et al., 2018; Zhang et al., 2019). Interestingly, *GmSWEET15* was not affected by inoculation with *S. sclerotiorum*, although its homologous gene *AtSWEET15* was downregulated after inoculation (Supplementary Figure S6; Supplementary Table S2A). This observation differed from the results of previous studies, however, *GmSWEET15* has also been identified as a susceptibility gene (Figures 1C, D), suggesting that *gmsweet15* mutant may adopt different strategies to resist infection with *S. sclerotiorum*.

WT and *gmsweet15* mutant upregulated defense response genes and inhibited photosynthesis at 24 hpi (Figures 3E, F, K, L, O, 4K). This is consistent with the results of many studies showing that plants inoculated with *S. sclerotiorum* activate the expression of defense response-related genes and downregulate the photosynthesis apparatus (Zhao et al., 2009; Ding et al., 2019; Ranjan et al., 2019; Sucher et al., 2020). Moreover, considering that enrichment analysis of DEGs depends only on the screening threshold, some important information may be lost. It is difficult to compare global expression patterns of certain biological processes under various conditions. Therefore, we combined the global defense response and photosynthesis genes to evaluate overall changes in trends. The results showed that *gmsweet15* mutant exhibited more pronounced transcriptional changes (Figures 3O, 4K).

*S. sclerotiorum* can manipulate the redox homeostasis of host plants using pathogenic factors such as oxalic acid to promote infection (Williams et al., 2011; Girard et al., 2017; Ding et al., 2020). Here, the WT downregulated cell redox homeostasis at 24 hpi, whereas no respective enrichment occurred in *gmsweet15* mutant. These results suggest that compared with WT, *S. sclerotiorum* easily destroyed the redox balance, making it in favor of colonization (Figure 3F). However, this process is likely associated with many obstacles that may have contributed to the failure of colonization in *gmsweet15* mutant (Figure 3L).

The MAPK signaling pathway is the key signal module downstream of the receptor/sensor that perceives either endogenous or exogenous stimuli, such as P/MAMPs and effectors, and it is involved in plant defenses against pathogenic fungi, including *S. sclerotiorum* (Zhang and Zhang, 2022). Moreover, several genes of MAPK cascades have been proved in genetic evidence that they positively regulate the resistance of plant to *S. sclerotiorum* (Wang et al., 2009, 2014, 2019c). Here, the WT and *gmsweet15* mutant upregulated this pathway at 24 hpi, which was more pronounced in *gmsweet15* mutant (Figures 3G, I, M, 4H). Reprogramming of the phenylpropanoid pathway is considered an important resistance characteristic of soybean resistance to *S. sclerotiorum* (Piasecka et al., 2015; Ranjan et al., 2019), and abundant antibacterial metabolites are biosynthesized to target ergosterol biosynthesis in fungi. Accordingly, *gmsweet15* mutant showed more substantial upregulation of this pathway (Figures 3G, J, M, 4J). These results imply that *gmsweet15* mutant exhibited faster responses and accumulated more secondary defense metabolites upon perceiving invasion by *S. sclerotiorum*.

Compared with WT, *gmsweet15* mutant showed more enriched biological processes at 24 hpi, such as upregulation of the secretory pathways and RAS/ARF protein signal transduction processes and inhibited tetrapyrrole metabolic process (Figures 3K, L). The mature processing and localization of PR1 seemed to be an important battlefield for pathogens in plants, and many effectors target this process (Yang et al., 2018; Sung et al., 2021; Li et al., 2022). In addition to being responsible for the mature processing and secretion of antibacterial peptides such as PR1, some endoplasmic reticulum and membrane transporters are associated with disease resistance capacity (Mao et al., 2017; Wang et al., 2021). Specific upregulation of secretion process may provide *gmsweet15* mutant with an advantage in confrontation with possible effectors (Figure 3K). The adenosine diphosphate ribosylation factor guanine nucleotide exchange factor protein family is involved in regulating various signal transduction processes and plant immunity (Lozano-Duran et al., 2014; Zwiewka et al., 2019; Zhao et al., 2020). Upregulation of the proteins of this family may contribute to *gmsweet15* mutant responses to *S. sclerotiorum* infection (Supplementary Figure S3E). Tetrapyrrole is closely related to chlorophyll degradation and leaf senescence, and it is an important retrograde signaling molecule that coordinates various stress responses by connecting chloroplasts with nucleus (Czarnecki et al., 2011; Barajas-Lopez Jde et al., 2013; Gao et al., 2016). Inhibition of tetrapyrrole metabolic process may lead to the accumulation of ROS in chloroplasts, but it does not exclude the effect of the retrograde signal from chloroplasts to nuclei, although the potential effects on interactions between soybean and *S. sclerotiorum* remain unclear. These biological processes of specific enrichment in *gmsweet15* mutant suggest that loss of *GmSWEET15* and superposition of the recognition of pathogens will specifically activate or inhibit these processes, which may play an important role in delaying early colonization by *S. sclerotiorum*. This also suggests that *GmSWEET15* may inhibit the response of these processes to infection with *S. sclerotiorum*, although the mechanisms remain elusive.

Ribosome biosynthesis typically affects plant growth and development (Saez-Vasquez and Delseny, 2019). Although some abiotic stress responses have been reported, ribosome biogenesis can adapt to these stresses by increasing the transcript levels of respective key genes. However, the role of ribosome biosynthesis in plant immunity has rarely been reported (Yu H. et al., 2020; Tieu Ngoc et al., 2021). In this study, compared to WT_24 h, *gmsweet15*_24 h upregulated the abundance of genes related to ribosome biosynthesis (Figures 4A, C). Several studies have shown that ribosomal biosynthesis and rRNA processing are involved in the sugar/glucose response (Kojima et al., 2007; Ishida et al., 2016; Maekawa et al., 2017). This implies that *GmSWEET15* loss may affect the communication between the nucleolar stress response pathway and the sugar response process during *S. sclerotiorum* infection, thus showing extraordinary upregulation of the ribosome biosynthesis pathway, compared with that in the WT. Ubiquitin regulates plant immunity in various ways, however, it has not been reported with regard to the interactions between plant and *S. sclerotiorum* (Dong et al., 2018; Wang J. et al., 2018; Ali et al., 2019; Feng et al., 2020; Liu et al., 2020; Gao et al., 2021). Here, the *gmsweet15* mutant upregulated ubiquitination more strongly, indicating that these ubiquitinated enzymes may also contribute to the resistance of *gmsweet15* mutant to *S. sclerotiorum* (Figure 4G). At present, it is inclined to think that plant immune response, especially MPK3/MPK6 activation, actively induces global downregulation of photosynthesis, thus coordinating the growth and defense trade-off (Bilgin et al., 2010; Ballare, 2014; Yang et al., 2014; de Torres Zabala et al., 2015). Moreover, photosynthetic inhibition is essential for the accumulation of ROS in chloroplasts and ETI activation (Su et al., 2018). Oxygen-evolving enhancer proteins have been implicated in stabilizing the photosystem, and their disruption leads to ROS accumulation in chloroplasts and enhances disease resistance (Lundin et al., 2007; Wang et al., 2019a). The rapid and drastic downregulation of these genes constituting the photosynthetic apparatus in *gmsweet15* mutant at 24 hpi suggests that the *gmsweet15* mutant may enhance its defense against *S. sclerotiorum* through higher intensity photoinhibition (Figure 4E). This intense transcriptional reprogramming may be an important strategy for *gmsweet15* mutant to resist *S. sclerotiorum*, which also implies that *GmSWEET15* loss may amplify the response of soybean to *S. sclerotiorum* in some ways.

According to WGCNA, the blue module was associated with high expression in *gmsweet15*_24 h and showed enriched calcium signaling and mediator complex processes (Supplementary Figure S5D; Figures 6A, D). Calcium influx is an important characteristic of plant immunity activation, and calcium signaling is involved in almost all aspects of plant immune signal transmission (Tian et al., 2020; Xu et al., 2022). The mediator complex also regulates the expression of defense genes through cooperative transcription complexes (Liao et al., 2016; Dolan et al., 2017; Mao et al., 2019; Agrawal et al., 2021; Chen et al., 2021; Kim et al., 2022). Considering the role of these two processes in plant immunity, it is obviously trustworthy for blue module to enrich them. Moreover, the genes involved in these two processes in the module are also candidate resistance to SSR genes. In addition, two hub genes, *Spg1* and *PTI1B*, were identified in the blue module (Figure 7A). Considering the generally induced expression of *Spg1* under various biotic and abiotic stresses (Guan and Nothnagel, 2004; Rizhsky et al., 2004; Sottosanto et al., 2007; Ascencio-Ibanez et al., 2008) and the role of *PTI1B* in plant immunity (Forzani et al., 2011), we propose that these hub genes are important candidates for resistance resources. Similarly, endoplasmic reticulum-related genes were enriched in the brown module (Figures 6F, G). Moreover, three endoplasmic reticulum proteins were identified in the 20 hub genes of this module (Figure 7B). This result further emphasizes the role of the endoplasmic reticulum in the response of *gmsweet15* mutant to *S. sclerotiorum* infection. ROS metabolism and peroxisome were enriched in the turquois module (Figures 6H, I), suggesting that *gmsweet15* may accumulate more ROS, which may be beneficial for blocking pathogen colonization. Collectively, we identified three modules related to the *gmsweet15* mutant phenotype, the correspondingly enriched biological processes and hub genes using WGCNA, which provided a reliable reference for subsequent selection of resistance resources.

## Conclusion

In the present study, through comparative transcriptome profiling, we preliminarily clarified the molecular mechanism of delaying the early colonization of *S. sclerotiorum* through more intense and extensive transcriptional reprogramming after the loss of *GmSWEET15*, and we propose numerous candidate functional genes for elevating SSR resistance.

## Data availability statement

The datasets presented in this study can be found in online repositories. The names of the repository/repositories and accession number(s) can be found in the article/Supplementary material.

## Author contributions

JL, KX, and KQ were responsible for experiment design and paper writing. SW, YX, and HP were responsible writing guidance. KQ, WC, XX, AL, XH, and ZS were responsible for experiment performance. KX, KQ, FW, and JL were responsible for experimental data processing and analysis. All authors contributed to the article and approved the submitted version.

## Funding

This work was supported by the National Natural Science Foundation of China (32172505), the Key Research and Development Program of Jilin Province (20210202131NC), and the Inter-Governmental International Cooperation Special Project of National Key R&D Program of China (2019YFE0114200).

## Conflict of interest

The authors declare that the research was conducted in the absence of any commercial or financial relationships that could be construed as a potential conflict of interest.

## Publisher’s note

All claims expressed in this article are solely those of the authors and do not necessarily represent those of their affiliated organizations, or those of the publisher, the editors and the reviewers. Any product that may be evaluated in this article, or claim that may be made by its manufacturer, is not guaranteed or endorsed by the publisher.

## Footnotes

^1^ http://www.broadinstitute.org/gsea/index.jsp
