# Supplementary figures and images for: Comparative transcriptome profiling reveals the importance of GmSWEET15 in soybean susceptibility to Sclerotinia sclerotiorum
Source: Front Microbiol. 2023 Jan 26;14:1119016. doi: 10.3389/fmicb.2023.1119016 (PMC9909833; doi:10.3389/fmicb.2023.1119016)

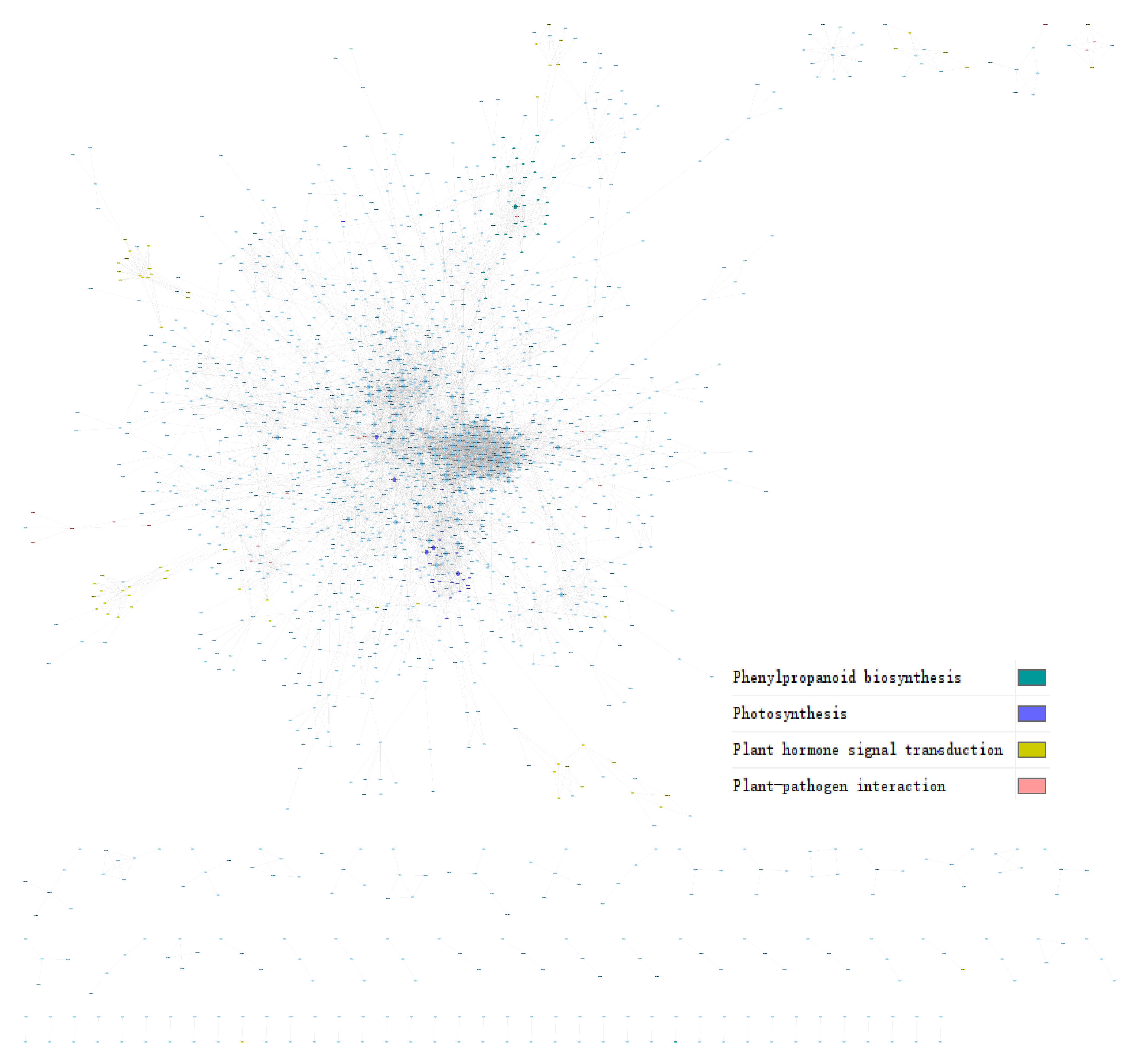

Supplement: Supplementary file 6 [file Image_6.TIF]
